# A Comparison of Methods for the Extraction of Plasmids Capable of Conferring Antibiotic Resistance in a Human Pathogen From Complex Broiler Cecal Samples

**DOI:** 10.3389/fmicb.2018.01731

**Published:** 2018-08-13

**Authors:** Sarah Delaney, Richard Murphy, Fiona Walsh

**Affiliations:** ^1^Antimicrobial Resistance and Microbiome Research Group, Department of Biology, Maynooth University, Maynooth, Ireland; ^2^Alltech European Bioscience Centre, Dunboyne, Ireland

**Keywords:** plasmids, extraction methods, broiler, antibiotic resistance, pathogen

## Abstract

The direct extraction of plasmid DNA containing antibiotic resistance genes from complex samples is imperative when studying plasmid-mediated antibiotic resistance from a One Health perspective, in order to obtain a wide representation of all the resistance plasmids present in these microbial communities. There are also relatively few bacterial species from natural environments which can be cultured *in vitro*. Extracting plasmids from the cultivable fraction of these complex microbiomes may only represent a fraction of the total antibiotic resistance plasmids present. We compared different methods of plasmid extraction from broiler cecal samples, whose resistance could be expressed in a human pathogen—*Escherichia coli*. We found that kits designed for DNA extraction from complex samples such as soil or feces did not extract intact plasmid DNA. Commercial kits specific for plasmid extraction were also generally unsuccessful, most likely due to the complexity of our sample and intended use of the kits with bacterial culture. An alkaline lysis method specific for plasmid extraction was ineffective, even with further optimization. Transposon-aided capture of plasmids (TRACA) allowed for the acquirement of a small range of resistance plasmids. Multiple displacement amplification provided the broadest range of resistance plasmids by amplifying all extracted circular plasmid DNA, but the results were not reproducible across all samples. Exogenous plasmid isolation enabled the extraction of resistance plasmids from the microbial fraction by relying on the mobility of the plasmids in the sample. This was the most consistent method from which we obtained a range of resistance plasmids from our samples. We therefore recommend the use of the exogenous plasmid isolation method in order to reliably obtain the greatest representation of the total antibiotic resistance plasmidome in complex samples. While this method has limitations, it is one which will vastly increase our current knowledge of antibiotic resistance plasmids present in complex environments and which are capable of transferring to a human and animal pathogen and environmental contaminant.

## Introduction

The rapid rise of antibiotic resistance has led to further studies into mobile genetic elements. Plasmids have been shown to be central vectors of gene sharing amongst bacteria (Halary et al., 2010), and therefore play a key role in microbial evolution and the spread of antibiotic resistance, leading to the rise of multi-resistant pathogenic bacteria (Tamminen et al., 2012). Bacterial plasmids allow resistance genes to transfer horizontally between taxa and between animals and humans (Sørensen et al., 2005). It is the mobility of these antibiotic resistance plasmids that is causing the most concern, as it is probably the most common mechanism for the dissemination of resistance genes (Perry et al., 2014), and many plasmids have the ability to move from a non-clinical environment to clinical pathogenic or human commensal bacteria.

To study the antibiotic resistance plasmidome of a microbial population, there must be efficient methods of extracting the plasmid population directly from the sample being examined. However, plasmids make up only a small proportion of the total DNA present in complex samples (Kav et al., 2013), and the cultivable component of the sample is even smaller. Traditional culture-based methods are less than ideal for working with animal or environmental samples as only a small fraction of these bacteria can be cultured in a laboratory environment (Kav et al., 2013). Therefore, a large proportion of the plasmids present in such samples are missed if relying solely on culture-based methods. Additionally, the use of metagenomics-based sequencing methods also has its limitations. The sequencing depth is usually insufficient to extract whole plasmids from the data, assembly is difficult due to the small size of the fragments, and genes present in low abundances are missed (Lynch and Neufeld, 2015). Also, plasmids often contain repeat sequences that are shared with genomic DNA, making assembly from short-read data difficult (Arredondo-Alonso et al., 2017). Therefore, there is a need to determine what methods are capable of extracting these resistance plasmids directly from complex samples and which will provide a wide representation of the antibiotic resistance plasmid population present in the microbial environment.

In this study, we examined six methods of plasmid extraction and used broiler cecal samples as representatives of complex samples. The gastrointestinal tract of broilers hosts a complex microbial community of hundreds of bacterial species (Oakley and Kogut, 2016). The plasmid DNA was transformed into *Escherichia coli* and selected on antibiotic plates to identify resistance plasmids. This allowed us to identify antibiotic resistance that could be expressed in a human pathogen, and further analyse the resistance mechanism in a well-characterized pathogen. There are also other plasmid extraction methods which have not been evaluated in this study but show good results. For example, Sentchilo et al. (2013) used a CsCl-EB method to isolate a variety of plasmids from activated sludge systems.

At present, there are no commercial kits designed to extract plasmid DNA directly from complex samples. Current plasmid extraction kits are intended to work with pure bacterial culture, which is less than ideal when dealing with complex environmental samples. Kits which are devised for use with complex samples such as soil or feces target only genomic DNA. Alkaline lysis (Birnboim and Doly, 1979) is a widely used method for the extraction of plasmid DNA by separating it from chromosomal DNA based on the small size and supercoiled nature of plasmids. However, it is also only intended for use with bacterial culture, not with complex samples which contain other material as well as bacteria.

Exogenous plasmid isolation works by capturing the plasmids directly from the complex sample in biparental matings using a recipient bacterium. While this method allows for plasmids to be obtained directly from the sample, it relies strongly on the plasmid being stably maintained in the host, and on the conjugative ability of the plasmids present in the sample. Therefore, this method may give a misrepresentation of the total plasmids present in the sample, as the non-conjugative fraction may not be extracted with this method (Dib et al., 2015). However, plasmids can become mobilized by a self-transmissible plasmid (Thomas and Nielsen, 2005), and could therefore also be captured by this method. Additionally, the exogenous method can in general also isolate linear plasmids, which are frequently found in diverse microbial environments (Dib et al., 2015).

The Transposon-aided capture (TRACA) of plasmids allows for the acquirement of antibiotic resistance plasmids from complex samples (Jones and Marchesi, 2007). It works by removing any contaminating chromosomal DNA from a total DNA sample, and then inserting a transposon onto the plasmids with a known selectable marker. Linear plasmids may not be captured by this method, as the *Tn5* origin of replication is not capable of replicating their extreme termini, and they could be degraded by the exonuclease unless specialized enzymes are used (Warburton et al., 2011). The main advantage of this method is that it has the capability to capture plasmids that do not have a selectable marker for *E. coli* and may not have the ability to replicate. It has been noted that this method favors the isolation of small plasmids, so it may give a misrepresentation of the total plasmid population (Jørgensen et al., 2015).

The multiple displacement amplification method works by removing all sheared genomic DNA from a total DNA sample with plasmid-safe DNase. The remaining circular plasmid DNA is amplified by phi29 DNA polymerase, which has a rolling-circle mechanism. In short, by using random hexamers, phi29 allows for the unspecific amplification of the circular plasmid DNA present. The benefit of this method is that even when plasmids are present in very small numbers compared to the total DNA in the sample, this method allows for the generation of large amounts of plasmid DNA (Kav et al., 2013). Similarly, this method also favors the selection of small plasmids (Jørgensen et al., 2015), and like TRACA, disregards linear plasmids, some of which could be degraded by DNase treatment (Dib et al., 2015). It should also be noted that large plasmids could be sheared during the extraction, by which they may also be degraded by the exonuclease treatment. Norman et al. (2014) described an electroelution step which could be applied prior to amplification to attempt to increase the number of large-sized plasmids obtained.

Our study compared these methods to identify which extracted the largest variety of antibiotic resistance plasmids (based on the banding patterns and resistance profiles of transformants or transconconjugants) present in the complex broiler cecal samples. We found that the exogenous isolation method best met these criteria, in both a time-efficient and consistent manner. While this method does not remove all bias, it does allow for the acquirement of antibiotic resistance plasmids which can be further phenotypically tested.

## Materials and methods

### Samples

The broiler cecal samples were collected from a commercial poultry production unit in the United Kingdom. Samples were lyophilized and stored at −80°C. Each of the plasmid extraction methods were carried out with the same cecal sample (Sample A). All methods were also carried out with *Escherichia coli* NCTC 13400 containing plasmid pEK499 as a control.

### Plasmid extractions and identifications

#### Culture dependent method

Cecal sample (0.01 g) was mixed with 0.1 mL of 0.85% NaCl. The 0.1 mL mix was spread on a non-selective Muller-Hinton (Merck) agar plate and incubated overnight at 37°C. All bacterial growth on the plate was scraped off, inoculated into 6 mL of Muller-Hinton broth and incubated overnight at 37°C with shaking at 225 rpm. Plasmid DNA was extracted from this bacterial culture using the Macherey-Nagel NucleoSpin Plasmid Kit according to the manufacturer's guidelines. The resulting DNA samples were visualized on a 1% agarose gel stained with GelRed (Biotium) and run at 70 volts (V) for 60 minutes (min).

#### Commercial DNA extraction kits

- DNA was extracted from 0.05 g of cecal sample using the Mobio PowerSoil DNA Extraction Kit (now Qiagen), according to the manufacturer's guidelines.
- DNA was extracted from 0.01 g of cecal sample using the Qiagen Plasmid Mini Kit, according to the manufacturer's guidelines.
- DNA was extracted from 0.01 g of cecal sample using the Macherey-Nagel NucleoSpin Plasmid Kit, according to the manufacturer's guidelines.

Extracted DNA was visualized on a 1% agarose gel stained with GelRed and run at 70 V for 60 min. The DNA was electroporated at 1.8 kV into *E. coli* DH5α, selected on ampicillin (32 mg/L), tetracycline (16 mg/L), kanamycin (25 mg/L), colistin (16 mg/L), and ciprofloxacin (4 mg/L), or incubated at 37°C overnight. Plasmid DNA was extracted from the transformants using the Machery-Nagel NucleoSpin Plasmid kit and digested with *Eco*RI restriction enzyme according to the manufacturer's guidelines. Plasmids were visualized on a 1% agarose gel stained with GelRed. Antibiotic susceptibility testing via the disk diffusion method was carried out on transformants according to the CLSI guidelines (Clinical Laboratory Standards Institute, 2016).

#### Alkaline lysis method

Plasmid DNA was extracted using an alkaline lysis method (Birnboim and Doly, 1979). The cecal sample (0.03 g) was resuspended in 100 μL ice-cold resuspension buffer [50 mM glucose, 25 mM TrisCl (pH 8.0), 10 mM EDTA (pH 8.0)]. Bacterial cells were lysed with 200 μL lysis solution [0.2 N NaOH, 1% (wt/vol) sodium dodecyl sulfate (SDS)] for 4 min and neutralized with 150 μL of chilled 3 M potassium acetate, pH 4.8. The samples were centrifuged at 14,000 rpm for 10 min at 4°C. The supernatant containing the plasmid was mixed with an equal volume of isopropanol and incubated at −20°C for 15 min. Samples were centrifuged at 14,000 rpm for 30 min at 25°C. The supernatant was removed and 500 μL of 70% ethanol was added to the pellet and centrifuged at 14,000 rpm for 5 min at 25°C. The pellet was resuspended in 50 μL MilliQ water. Extracted DNA was visualized on a 1% agarose gel stained with GelRed and run at 70 V for 60 min. The DNA was electroporated into *E. coli* DH5α, selected on ampicillin (32 mg/L), tetracycline (16 mg/L), kanamycin (25 mg/L), colistin (16 mg/L), or ciprofloxacin (4 mg/L), and incubated at 37°C overnight. Plasmid DNA was extracted from the transformants using the Machery-Nagel NucleoSpin Plasmid kit and digested with *Eco*RI restriction enzyme. Plasmids were visualized on a 1% agarose gel stained with GelRed run at 70 V for 60 min. Antibiotic susceptibility testing via the disk diffusion method was carried out on transformants according to CLSI guidelines.

#### Exogenous plasmid isolation

Plasmid DNA was exogenously isolated in biparental matings (Kyselková et al., 2016). Cecal sample (0.01 g) was added to 0.9 mL of non-selective Tryptic Soy Broth (TSB) (Sigma Aldrich) and incubated at 20°C on a rocker at 70 rev/min overnight. The supernatant containing the bacterial fraction (0.8 mL) was centrifuged at 2,800 × g for 10 min at room temperature (RT). The pellet was resuspended in 80 μL of TSB. This comprised the donor culture. A culture of rifampicin resistant *E. coli* DH5α was grown overnight at 28°C and shaking at 180 rpm. The bacterial content was pelleted by centrifugation at 2,800 × g for 5 min at RT, washed in 140 μL LB broth (Duchefa-Biochemie) and resuspended in 140 μL LB broth. This comprises the recipient culture. Donor and recipient culture (50 μL each) were mixed and centrifuged at 2,800 × g for 5 min at RT. The supernatant was removed and the pellet resuspended in 50 μL of TSB. This was applied to a 0.2 μm filter on an LB agar plate and incubated at 28°C for 20 h. The filter was removed from the plate and the cells resuspended in 0.85% NaCl by vortexing. A volume of 100 μL was plated on Eosin Methylene Blue (EMB) agar (Sigma) with rifampicin (100 mg/L) and one of the following antibiotics: ampicillin (32 mg/L), tetracycline (16 mg/L), kanamycin (25 mg/L), colistin (16 mg/L), or ciprofloxacin (4 mg/L). Plates were incubated at 28°C for 1–2 days until transconjugant colonies appeared. Plasmids were extracted from each of the colonies using the Macherey-Nagel NucleoSpin Plasmid Kit and digested with *Eco*RI restriction enzyme. Plasmids were visualized on a 1% agarose gel stained with GelRed run at 70 V for 60 min. Antibiotic susceptibility testing via the disk diffusion method was carried out on transconjugants according to CLSI guidelines.

#### Transposon-aided capture of plasmids (TRACA)

Transposon-Aided Capture of plasmids (TRACA) was carried out as previously described (Jones and Marchesi, 2007). TRACA is based on the insertion of a transposon with a known origin of replication and antibiotic resistance marker into the plasmids, which can then subsequently be “captured.” Bacterial cells were separated from the cecal samples by adding 0.1 g of cecal sample to 0.9 mL of non-selective TSB and incubating at 20°C and on a rocker at 70 rev/min overnight. The supernatant (0.8 mL) was centrifuged at 2,800 × g for 10 min at RT and DNA was extracted by performing alkaline lysis (as previously described) on the pellet.

The removal of sheared chromosomal DNA prior to performing the TRACA reaction ensures that the transposon is only inserted onto plasmid DNA. DNA was treated with Plasmid-Safe DNase (Epicenter), according to the manufacturer's guidelines. Amplification of the 16S rRNA genes by PCR was performed to ensure the ratio of plasmid:chromosomal DNA in the sample was reversed. This was carried out using the following primers: Forward 5′- TCGTCGGCAGCGTCAGATGTGTATAAGAGACAGCCTACGGGNGGCWGCAG-3′ and Reverse 5′- GTCTCGTGGGCTCGGAGATGTGTATAAGAGACAGGACTACHVGGGTATCTAATCC-3′; (Klindworth et al., 2013) and under the following conditions: 95°C for 3 min; 35 cycles of: 95°C for 30 s, 55°C for 30 s, 72°C for 30 s; and finally 72°C for 5 min.

TRACA was performed using the EZ-*Tn5* <R6Kγori/KAN-2> Insertion Kit (Epicenter), according to the manufacturer's guidelines. The 50 μL reaction was diluted with 450 μL sterile water and purified with Vivaspin 500 MWCO 100,000 Protein Concentrator Spin Columns (GE Healthcare Life Sciences) which reduced the reaction volume to 10 μL. Five microliters was electroporated at 1.8 kV into 100 μL TransforMax EC100D pir-116 Electrocompetent *E. coli* (Epicenter). The transformed cells were spread onto LB agar plates with 50 mg/L kanamycin to select for EZ-*Tn5*. Plasmid DNA was extracted from TRACA clones using the Qiagen HiSpeed Plasmid Midi kit and visualized on a 1% agarose gel stained with GelRed, run at 70 V for 60 min. Bands of plasmid DNA (B1 and B2) were harvested from a 1% agarose gel stained with SYBR Safe using the Cleaver Scientific runVIEW system run at the same conditions as before. The harvested DNA bands were electroporated into *E. coli* DH5α, selected on ampicillin (32 mg/L), tetracycline (16 mg/L), kanamycin (25 mg/L), colistin (16 mg/L), or ciprofloxacin (4 mg/L), and incubated at 37°C overnight. Transformants were obtained on ampicillin, tetracycline, and ciprofloxacin with DNA from band 2 and on ciprofloxacin with DNA from band 1. Plasmid DNA was extracted from the transformants using the Machery-Nagel NucleoSpin Plasmid kit and digested with *Eco*RI restriction enzyme. Plasmids were visualized on a 1% agarose gel stained with GelRed and run at 70 V for 60 mins. Antibiotic susceptibility testing via the disk diffusion method was carried out on transformants according to CLSI guidelines.

#### Multiple displacement amplification

The multiple displacement amplification method utilizes the rolling circle amplification mechanism of phi29 DNA polymerase to obtain large amounts of plasmid DNA from a complex sample. Plasmid DNA was extracted from the cecal sample by following protocol B (Kav et al., 2013), which was adapted from Hansen and Olsen (1978). The cecal sample (0.225 g) was resuspended in 8.1 mL of 25% sucrose, 50 mM Tris (pH 8). Lysozyme [10 mg/mL in 250 mM Tris (pH 8)] (0.6 mL) was added and the reaction was incubated on ice for 5 min. EDTA (3 mL of 250 mM, pH 8) was added and incubated on ice for 5 min. Sodium dodecyl sulfate (SDS) (6 mL of 10%) was added and mixed by inversion. Samples were incubated for eight cycles of heat pulsing and mixing (15 s at 55°C, 15 s at RT). NaOH (3 mL of 3 M) was added and mixed by inversion for 3 min. Tris (6 mL of 2 M, pH 7.0) was added and mixed by inversion. SDS (7.92 mL of 10%) was added, followed immediately by 7.5 mL of 5 M NaCl. Samples were incubated at 4°C overnight. Samples were centrifuged at 3,000 × g for 30 min at 4°C and the supernatant transferred to a new tube. 0.1 volume of 3 M sodium acetate (pH 5.2) and 0.6 volume of isopropanol were added and samples incubated overnight at 4°C.

As with TRACA, sheared chromosomal DNA was removed with plasmid-safe DNase prior to amplification to ensure only circular plasmid DNA was amplified. Removal of chromosomal DNA and amplification of plasmid DNA was carried out as described previously by Kav et al. (2013). A 50 μL reaction composed of 20 μL DNA, 24 μL MilliQ water, 1 μL ATP, 2.5 μL reaction buffer, and 2.5 μL plasmid-safe DNase was incubated at 37°C overnight and deactivated at 70°C for 30 min. Amplification of the 16S rRNA genes by PCR as previously described was performed to ensure the ratio of plasmid:chromosomal DNA is was reversed in the sample, i.e., high plasmid to low chromosomal DNA ratio. If bands were visible the assay was repeated. 0.1 volumes of 3 M sodium acetate (pH 5.2) and 0.6 volumes of isopropanol were added and incubated overnight at 4°C. Samples were centrifuged at 14,000 rpm 4°C for 30 min. The supernatant was removed and 70% ethanol added. Samples were mixed and centrifuged at 14,000 rpm 4°C for 15 min. The supernatant was removed and the pellet resuspended in 10 μL MilliQ water.

Plasmid DNA was amplified by adding 1 μL of 10 μM Exo-Resistant Random Primer (Thermo Scientific), 2 μL phi29 DNA Polymerase Reaction Buffer (New England Biolabs) and 8.2 μL of MilliQ water to 5 μL of the purified treated DNA. Samples were incubated at 95°C for 5 min and immediately chilled on ice for 5 min. 1.6 μL phi29 DNA polymerase (New England Biolabs), 0.02 μL of inorganic pyrophosphatase (from yeast) (New England Biolabs) and 2 μL of dNTPs (10 mM) (Thermo Scientific) were added and incubated at 30°C for 16 h.

Amplified plasmid DNA (5 μL) was electroporated at 1.8 kV into 15 μL of *E. coli* DH5α cells. Transformants were plated on LB agar plates with one of the following antibiotics: ampicillin (32 mg/L), tetracycline (16 mg/L), kanamycin (25 mg/L), cefotaxime (16 mg/L), colistin (16 mg/L), ciprofloxacin (16 mg/L). Plasmids were extracted using the Qiagen HiSpeed Midi kit and digested with *Eco*RI restriction enzyme. Plasmids were visualized on a 1% agarose gel stained with GelRed run at 70 V for 60 mins. Antibiotic susceptibility testing via the disk diffusion method was carried out on transformants according to CLSI guidelines.

## Results

### Culture dependent method

**Figure 1:**
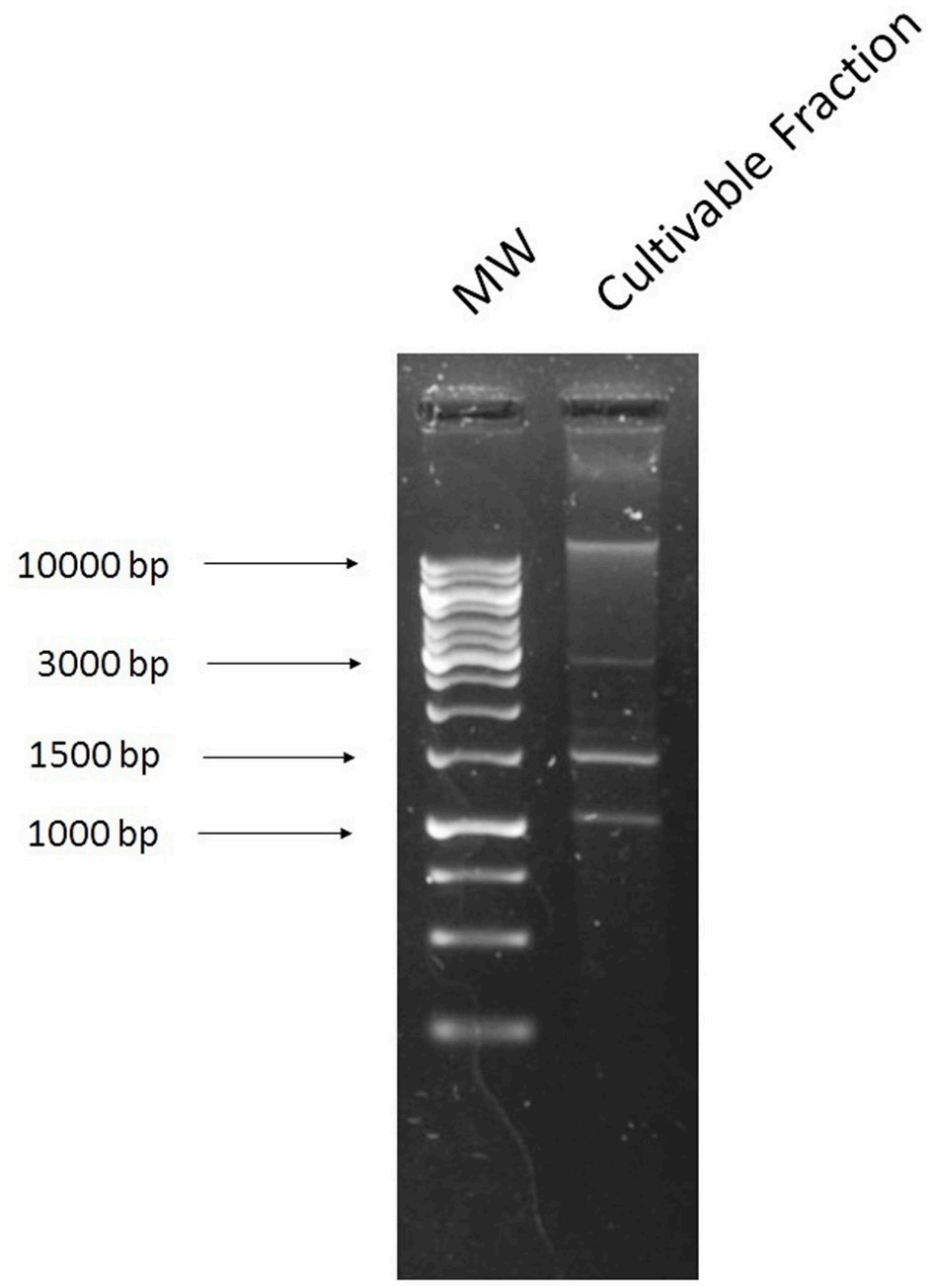
Agarose gel image of the plasmids extracted using the culture-dependent method from the broiler cecal sample, which was grown on non-selective Mueller-Hinton media and extracted with the Macherey-Nagel NucleoSpin Plasmid kit. **1**, 1 kb ladder; **2**, DNA extracted from the cultivable fraction of the cecal sample.

All cultivable bacteria grew on a non-selective rich medium and the DNA was extracted using a commercial plasmid extraction kit. Several bands were visible on an agarose gel (Figure 1), however when transformation was carried out it failed to yield any transformants on antibiotic plates. This could indicate that the plasmids present in the cultivable fraction did not harbor any resistance genes to the antibiotics tested. The plasmid pEK499 in *E. coli* was used as a pure bacterial culture control, and was successfully extracted using this method (Supplementary Figure 1).

### Commercial DNA extraction kits

**Figure 2:**
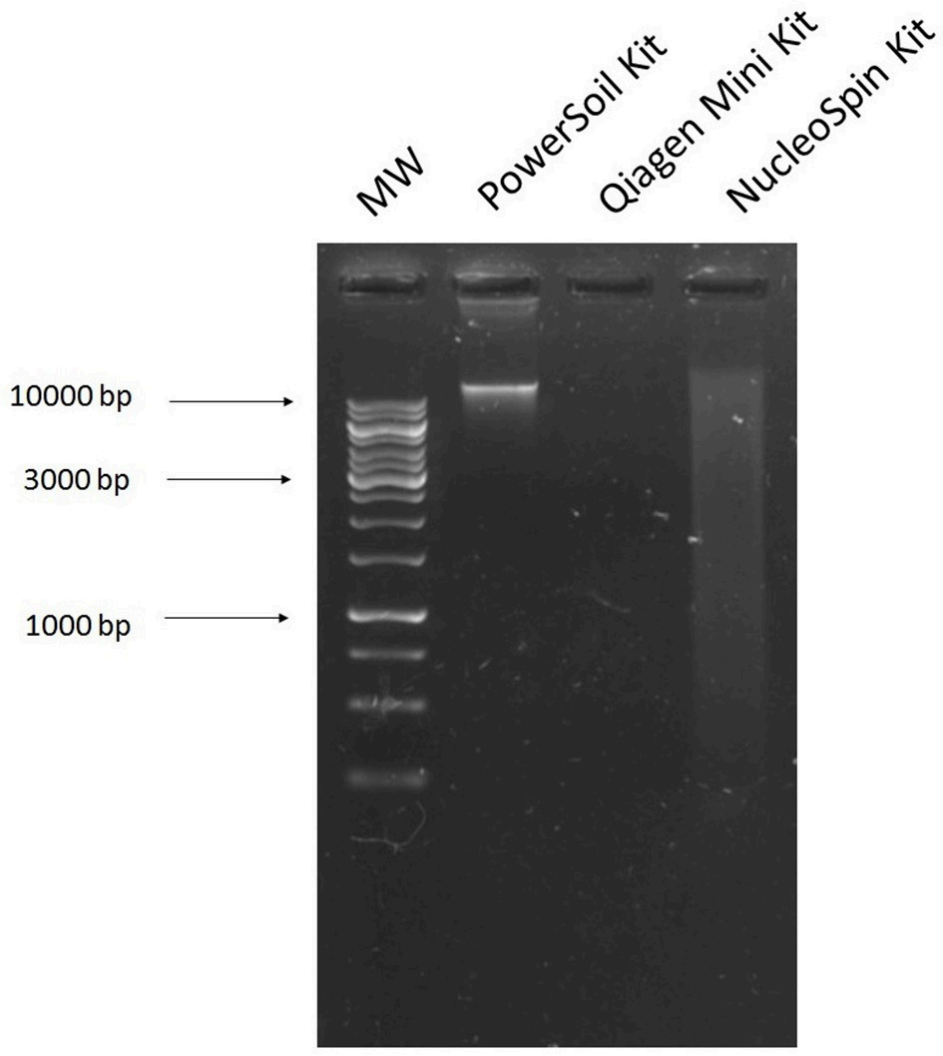
Agarose gel image of DNA extracted from cecal samples using commercial kits. **1**, 1 kb ladder; **2**, DNA extracted from the cecal sample using the MoBio PowerSoil DNA Isolation Kit; **3**, DNA extracted from the cecal sample using the Qiagen Plasmid Mini Kit; **4**, DNA extracted from the cecal sample using the Macherey-Nagel NucleoSpin Plasmid Kit.

The MoBio kit resulted in a single band of DNA located near the top of the agarose gel (Figure 2). Initially we thought that this band was genomic DNA or large plasmids. However, as no transformants were obtained after electroporation on any antibiotic plates [ampicillin (32 mg/L), tetracycline (16 mg/L), kanamycin (25 mg/L), colistin (16 mg/L), and ciprofloxacin (4 mg/L)] we concluded that this was genomic DNA. We also used this kit with 5 ml of *E. coli* culture harboring our control plasmid pEK499, which resulted in a very bright band (Supplementary Figure 2). It appears that as the DNA is at such a high concentration, and pEK499 is a large plasmid which diffuses slowly through the agarose gel, it is likely present along with genomic DNA.

The Qiagen Plasmid Mini kit and the Machery-Nagel NucleoSpin Plasmid kit are both designed for the extraction of plasmids from bacterial culture. Both kits work well for this purpose, which can be seen in Supplementary Figure 2, where they both extracted our control plasmid pEK499 from *E. coli* culture. However, when we used these kits with our cecal sample, we did not obtain clear bands of plasmid DNA. The NucleoSpin kit resulted in a smear on the gel (Figure 2), and yielded transformants on ciprofloxacin and tetracycline selective plates only. After subjecting these tranformants to a further plasmid extraction, digestion and antibiotic susceptibility testing, they had the same banding pattern and resistance profile. The Qiagen plasmid kit did not appear to retrieve any DNA from our samples (Figure 2) and did not yield any transformants on any antibiotic selective plates [ampicillin (32 mg/L), tetracycline (16 mg/L), kanamycin (25 mg/L), colistin (16 mg/L), and ciprofloxacin (4 mg/L)].

**Figure 3:**
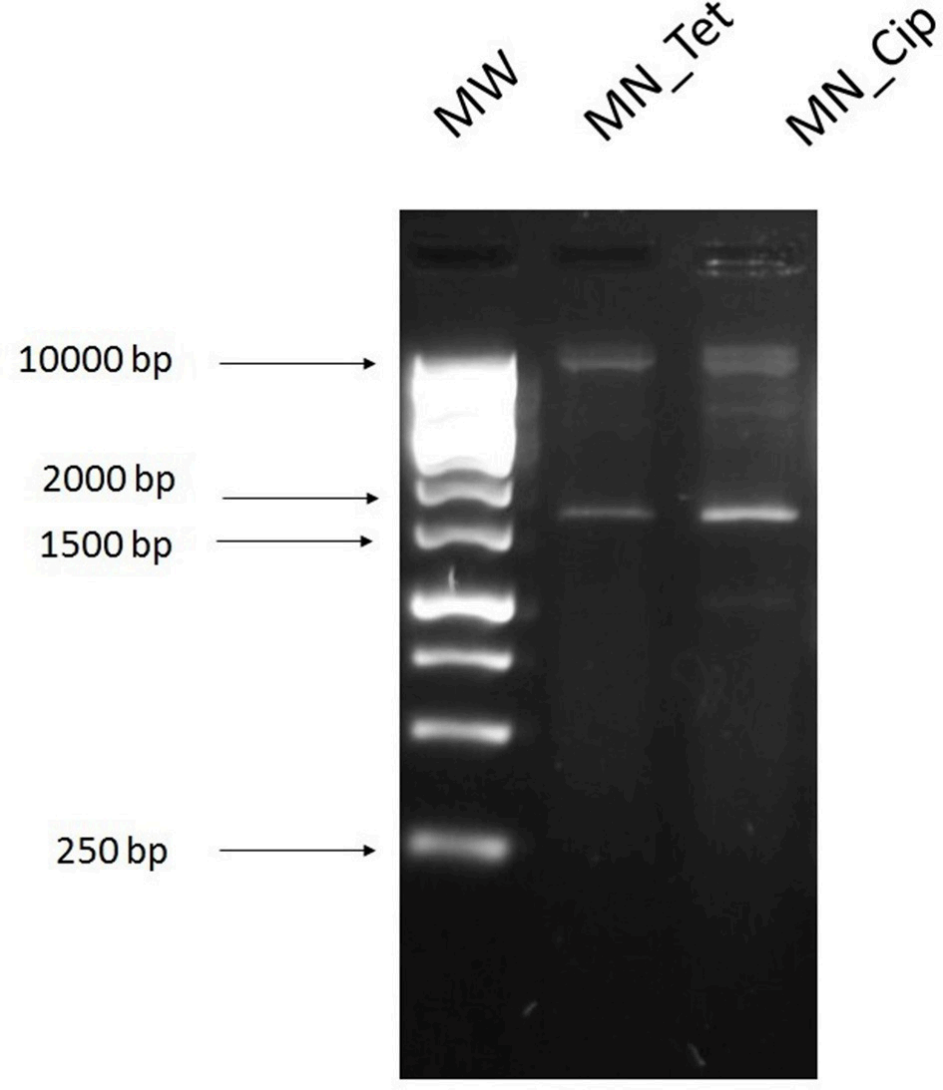
Agarose gel image of digested plasmid DNA extracted from transformants, which were obtained by electroporating the DNA from the direct extraction with the Macherey-Nagel NucleoSpin Plasmid kit into *E. coli* and digested with *Eco*RI restriction enzyme. **1**, 1 kb ladder; Digested plasmid DNA extracted from transformants selected on agar plates containing **2**, tetracycline 16 mg/L (MN_Tet); **3**, ciprofloxacin 4 mg/L (MN_Cip).

Plasmid DNA extracted from the cecal sample using the Macherey-Nagel NucleoSpin Plasmid kit was transformed into *E. coli* DH5α. Transformants grew on ciprofloxacin (4 mg/L) and tetracycline (16 mg/L) plates only (Figure 3). Antibiotic susceptibility testing via a disk diffusion provided the resistance profile of the resulting transformants (Table 1).

**Table 1:**
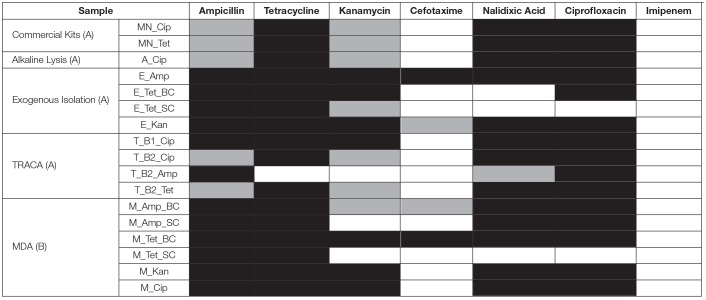
Disk diffusion results of resistant transformants obtained from each of the extraction methods.

*Black, Resistant; Gray, Intermediate; White, Susceptible; determined according to the (Clinical Laboratory Standards Institute, 2016)*.

*MN, Machery Nagel kit; A, Alkaline Lysis; E, Exogenous Isolation; T, TRACA; M, MDA*.

*Amp, selected on ampicillin; Tet, selected on tetracycline; Kan, selected on kanamycin; Cip, selected on ciprofloxacin*.

*B1, Lower band on gel; B2, Higher band on gel; both extracted and electroporated into E. coli DH5a*.

*(BC) And (SC) refer to the two different colony morphologies, big or small colonies, present on the same antibiotic plate*.

### Alkaline lysis method

**Figure 4:**
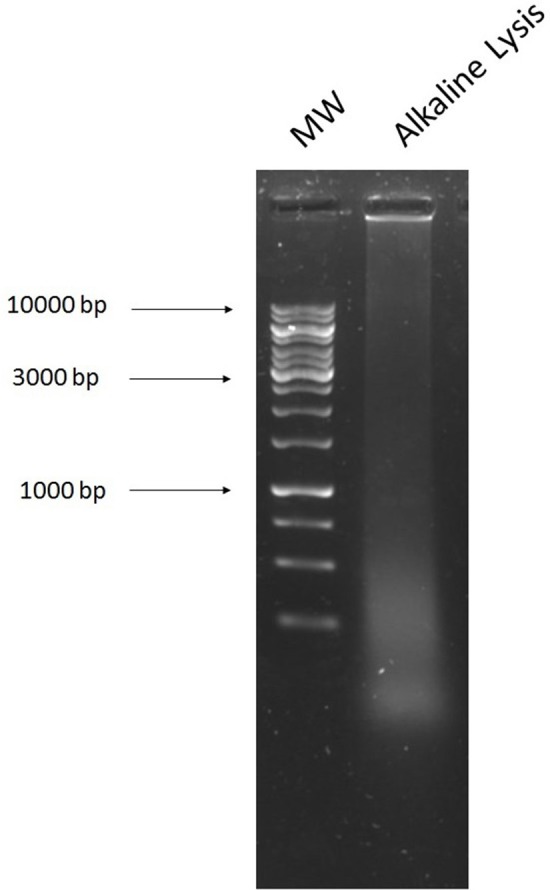
Agarose gel image of DNA extracted from the cecal sample using the alkaline lysis method. **1**, 1 kb Ladder; **2**, DNA extracted from the cecal sample using the alkaline lysis method.

**Figure 5:**
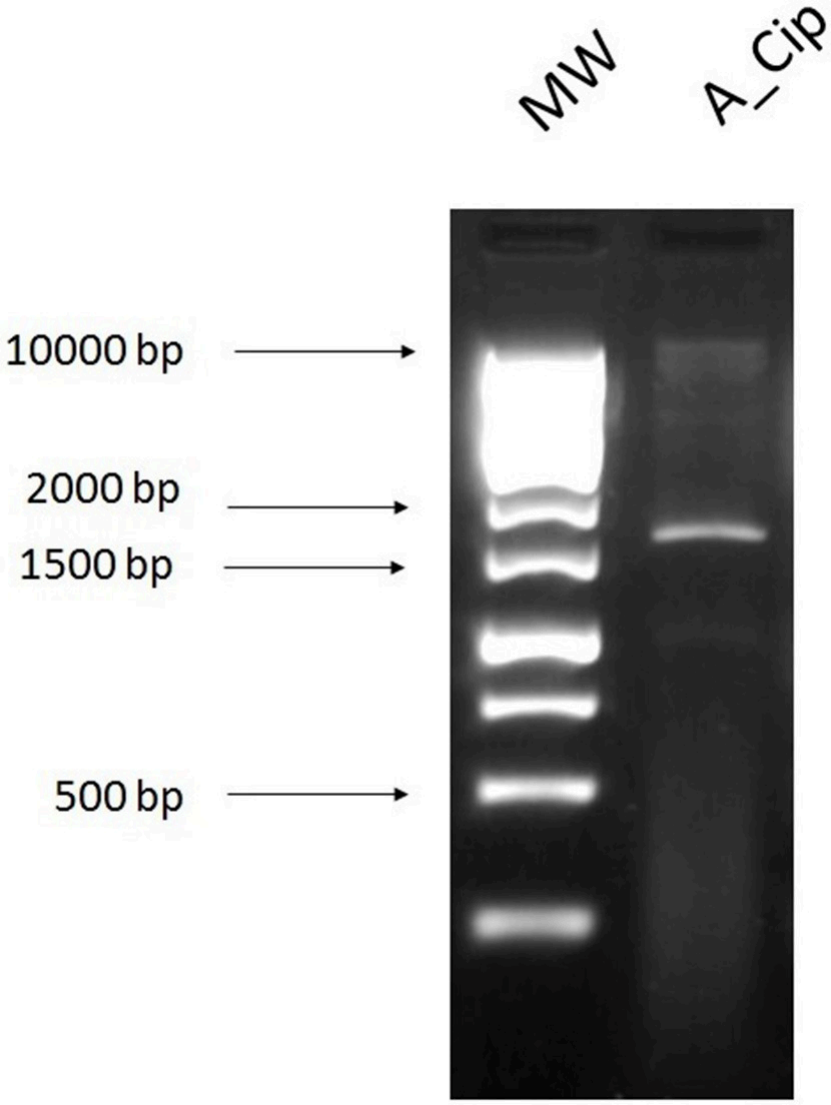
Agarose gel image of digested plasmids which were obtained by transforming *E. coli* with plasmid DNA extracted using the alkaline lysis method and selected on ciprofloxacin 4 mg/L (A_Cip) and digested with *Eco*RI. **1**, 1 kb ladder; **2**, digested plasmid DNA extracted from the transformant and selected on ciprofloxacin 4 mg/L (A_Cip).

A smear of DNA on a gel was detected after performing alkaline lysis directly on the cecal sample (Figure 4). This was the best result, even after reducing the sample volume (0.03 g), adding additional bead beating steps at varying time lengths, and the addition of varying concentrations of RNase A, proteinase K and lysozyme at different time points and incubation temperatures. We obtained transformants on ciprofloxacin (4 mg/L), which had similar banding patterns (Figure 5) and resistance profiles to the transformants selected on ciprofloxacin obtained with the NucleoSpin kit. The extracted DNA was electroporated into *E. coli*, selected on ciprofloxacin (4 mg/L), extracted from the transformants and digested with *Eco*RI (Figure 5). The method was repeated with control plasmid pEK499 (Supplementary Figure 3).

### Exogenous plasmid isolation

**Figure 6:**
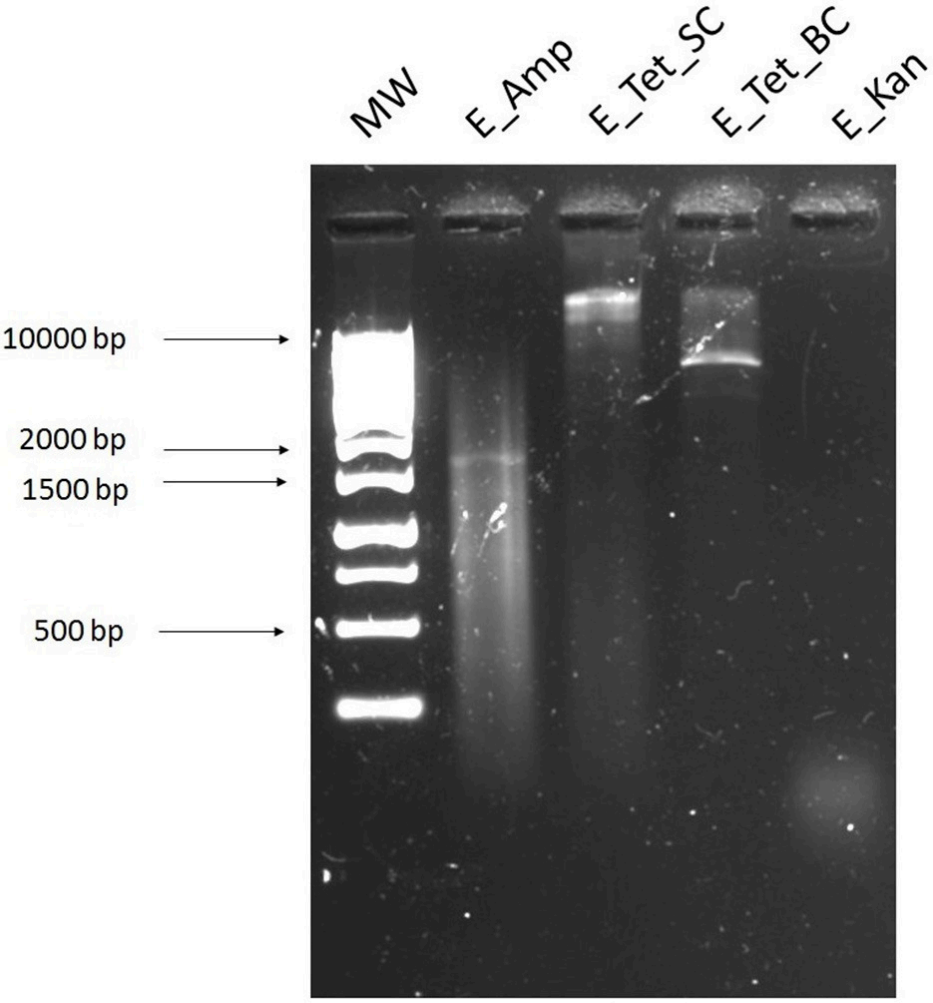
Agarose gel image of exogenously isolated plasmids from the cecal sample digested with *Eco*RI restriction enzyme. **1**, 1 kb ladder and DNA extracted from the cecal sample using the exogenous plasmid isolation method. Digested plasmid DNA extracted from transformants selected on agar plates containing **2**, ampicillin 32 mg/L (E_Amp); **3**, tetracycline (SC) 16 mg/L (E_Tet_SC); **4**, tetracycline (BC) 16 mg/L (E_Tet_BC); and **5**, kanamycin 25 mg/L (E_Kan). BC and SC refer to the two different colony morphology types, big or small colonies, on the same antibiotic plate.

The exogenous plasmids were obtained by the recipient in biparental matings, and selected on ampicillin (32 mg/L), tetracycline (16 mg/L), kanamycin (25 mg/L), colistin (16 mg/L), and ciprofloxacin (4 mg/L). Transformants were isolated from the plates containing ampicillin, tetracycline [with two colony morphologies: big colonies (BC) and small colonies (SC)], and kanamycin. This method isolated plasmids obtained from the cecal sample and control plasmid pEK499 (Figure 6; Supplementary Figure 4).

### Transposon-aided capture of plasmids (TRACA)

**Figure 7:**
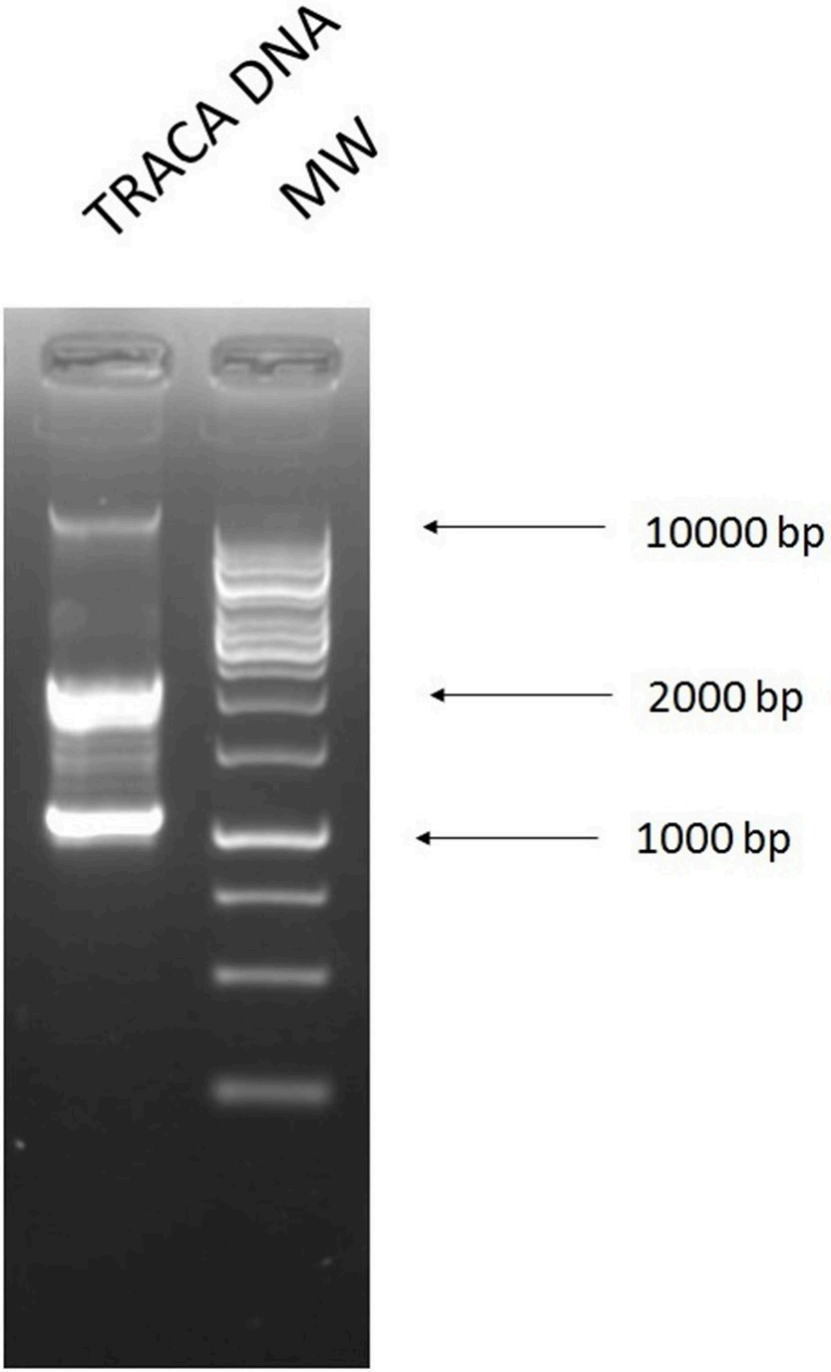
DNA extracted from cecal sample using the TRACA method of plasmid isolation. **1**, DNA extracted from transformants selected on kanamycin 50 mg/L after TRACA reaction and **2**, 1 kb ladder.

**Figure 8:**
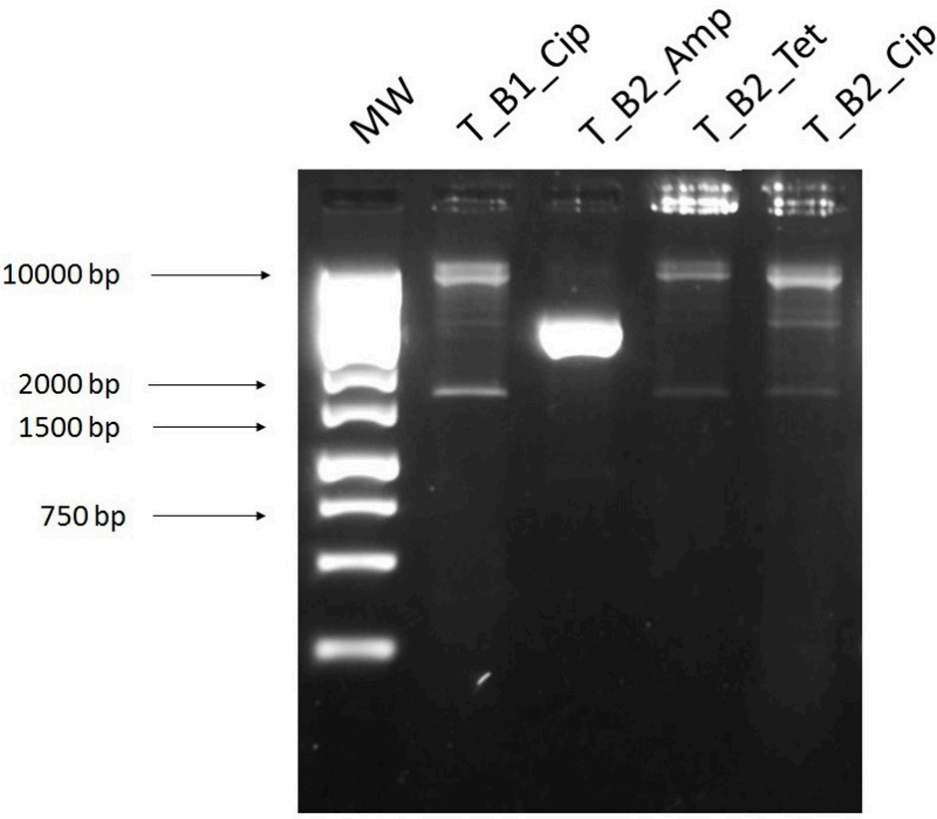
Digested plasmid DNA extracted from *E. coli* transformed with plasmid DNA from TRACA clones and selected on antibiotics. **1**, 1 kb ladder and bands of plasmid DNA extracted from a 1% SYBR safe gel and transformed into *E. coli*. Digested plasmid DNA extracted from transformants selected on agar plates containing; **2**, B1 ciprofloxacin 4 mg/L (T_B1_Cip); **3**, B2 ampicillin 32 mg/L (T_B2_Amp); **4**, B2 tetracycline 16 mg/L (T_B2_Tet) and **5**, B2 ciprofloxacin 4 mg/L (T_B2_Cip).

TRACA allowed for the acquisition of plasmids from the cecal samples by inserting a transposon with a selectable resistance marker and transforming the DNA into *E. coli*. The two largest bands of plasmid DNA were extracted directly from the gel (Figure 7) (B1, lower band; B2, higher band) and electroporated into *E. coli*. Transformants were selected on ampicillin (32 mg/L), tetracycline (16 mg/L), kanamycin (25 mg/L), colistin (16 mg/L), and ciprofloxacin (4 mg/L), with ampicillin, tetracycline, and ciprofloxacin plates yielding transformants (Figure 8). We found that all but one of the transformants tested had a similar banding pattern and resistance profile to the plasmids extracted using the alkaline lysis method and NucleoSpin kit.

### Multiple displacement amplification

**Figure 9:**
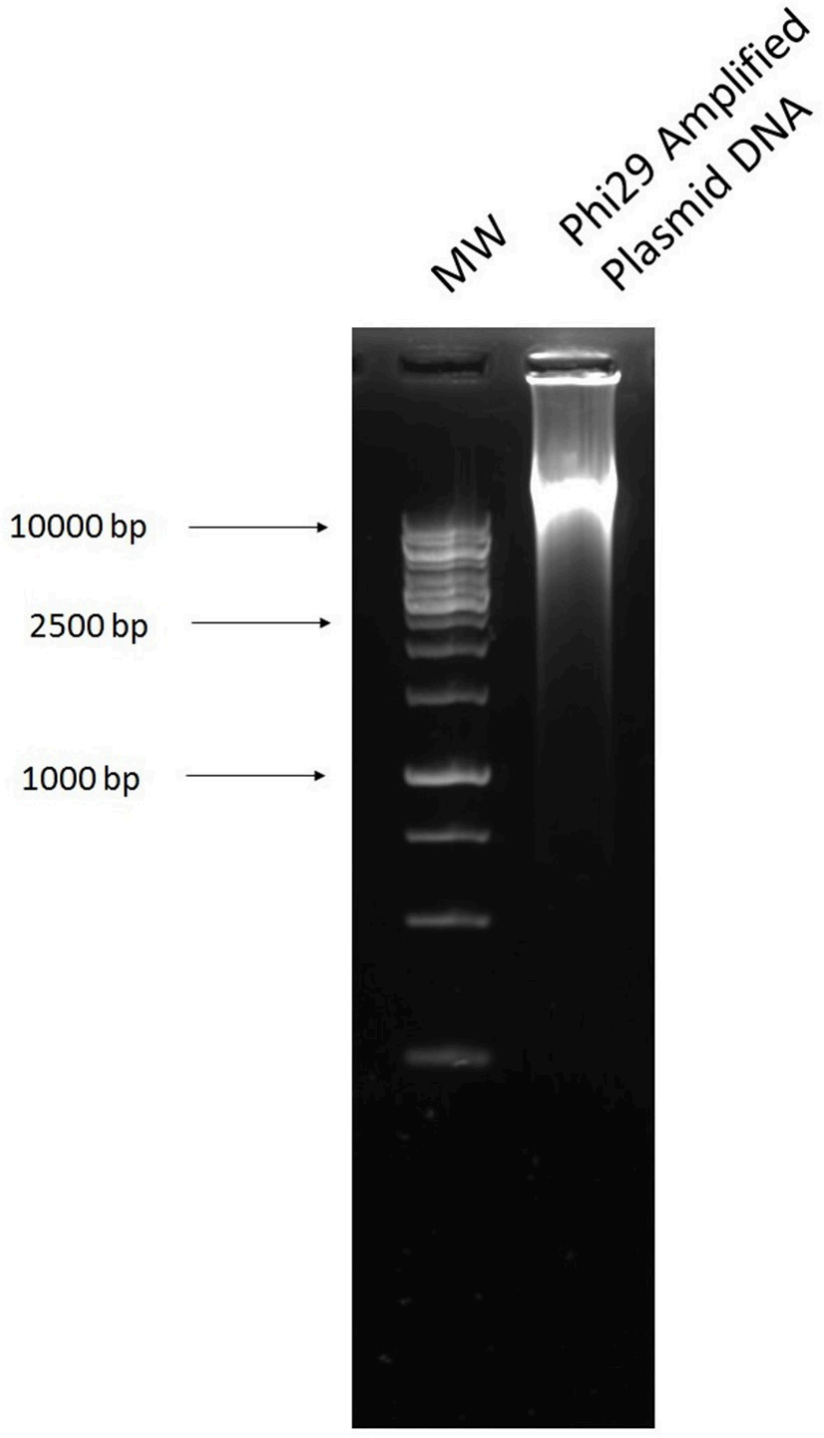
Plasmid DNA from the cecal sample after amplification with phi29 polymerase. **1**, 1 kb ladder and **2**, Plasmid DNA amplified with Phi29 DNA polymerase.

**Figure 10:**
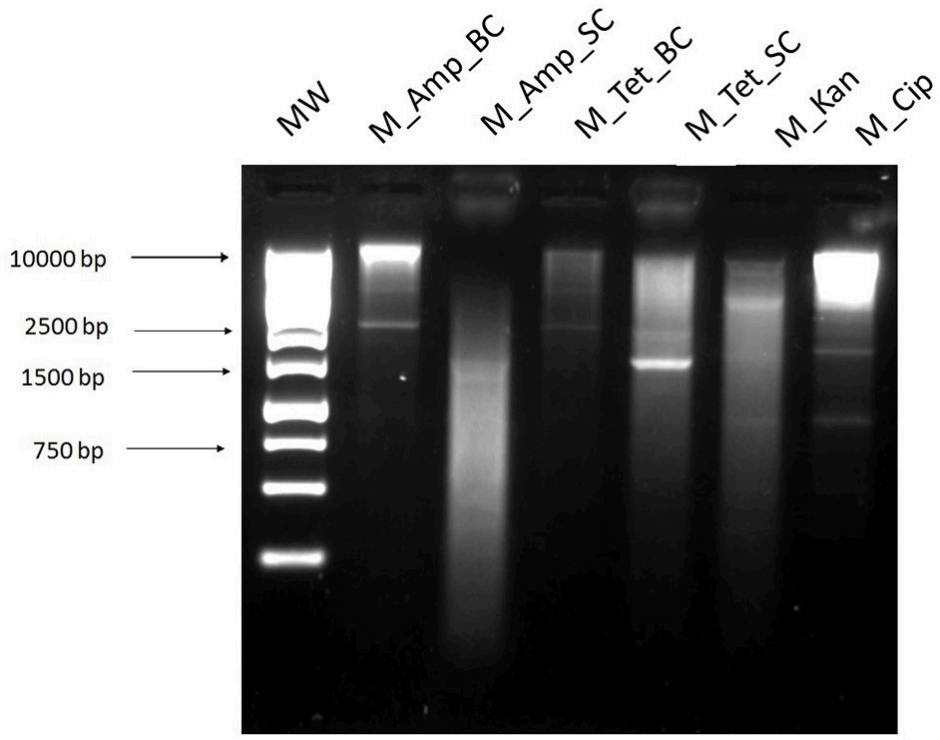
Digested plasmid DNA extracted from *E. coli* transformants after electroporation with the phi29 polymerase amplified DNA. **1**, 1 kb ladder; Plasmid DNA extracted from transformants selected on agar plates containing; **2**, ampicillin 32 mg/L (M_Amp_BC); **3**, ampicillin 32 mg/L (M_Amp_SC); **4**, tetracycline 16 mg/L (M_Tet_BC); **5**, tetracycline 16 mg/L (M_Tet_SC); **6**, kanamycin 25 mg/L (M_Kan); **7**, ciprofloxacin 16 mg/L (M_Cip). BC and SC refer to the two different colony morphology types, big or small colonies, on the same antibiotic plate.

The multiple displacement amplification method allows for unspecific but selective amplification of circular DNA after DNase digestion (Figure 9), through which we acquired antibiotic resistance plasmids from a cecal sample. This method gave us the largest range of plasmids from our cecal samples. That is, the greatest number of antibiotic plates [ampicillin (32 mg/L), tetracycline (16 mg/L), kanamycin (25 mg/L), and ciprofloxacin (16 mg/L)] which yielded transformants and each antibiotic plate transformants had a different banding pattern after digestion with *Eco*RI (Figure 10) and resistance profile (Table 1). However, the results shown are from a different cecal sample (Sample B) as the method was unsuccessful for sample A.

**Figure 11:**
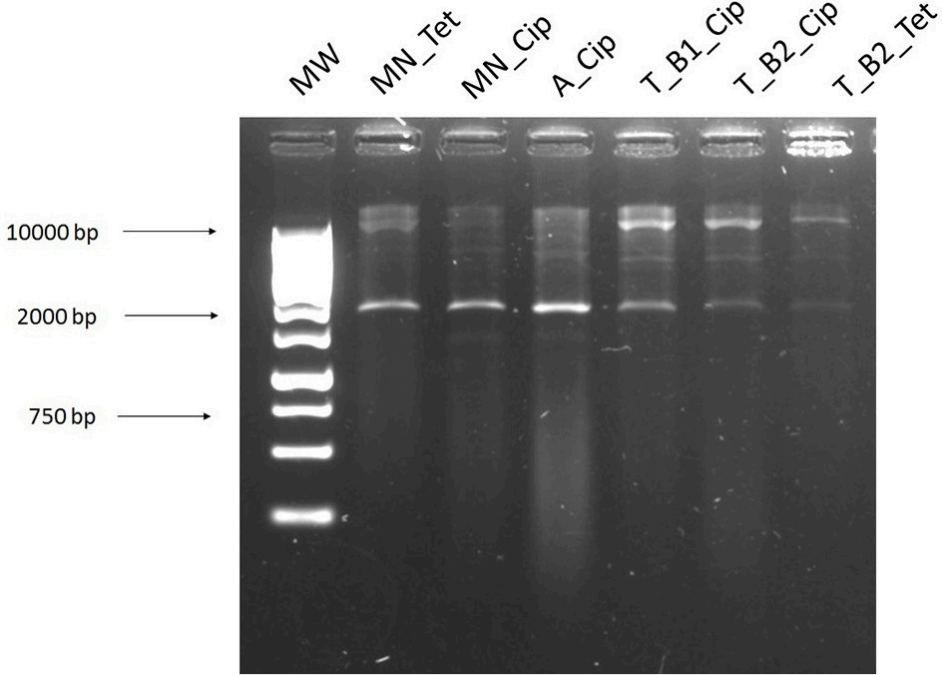
Transformants from different isolation methods with identical banding patterns after plasmid digestion. Along with similar resistance profiles, this indicates the strains are probably harboring the same plasmid. **1**, 1 Kb Ladder; **2**, MN_Tet; **3**, MN_Cip; **4**, A_Cip; **5**, T_B1_Cip; **6**, T_B2_Cip; **7**, T_B2_Tet.

Antibiotic susceptibility testing via a disk diffusion method gave the resistance profile of the plasmids (Table 1). This, along with the banding patterns of the digested plasmids on agarose gels, allowed for the identification of the variety of plasmids obtained from each extraction method. It also allowed for a comparison of the plasmids acquired using the different methods from the same sample, to determine if the same or different plasmids were obtained. There was no single antibiotic that selected for transformants using all methods. However, plasmids with identical antibiotic resistance patterns were identified using the different methods. Transformants isolated using the exogenous method had four different antibiotic susceptibility patterns, suggesting the presence of at least four different plasmids. The exogenous transformants had the widest range of resistance, with three transformants resistant to four different classes of antibiotics. T_B1_Cip transformant had the same resistance profile as M_Kan and M_Cip selected transformants. Based on visual analysis of the banding patterns (Figure 11) and antimicrobial susceptibility patterns (Table 2) combined, the plasmids identified in MN_Cip, MN_Tet, A_Cip, T_B2_Cip, and T_B2_Tet are probably the same plasmids or highly similar. Further analysis methods, such as sequencing, are required to confirm that these plasmids are identical. The remaining transformants had unique resistance profiles.

**Table 2:**
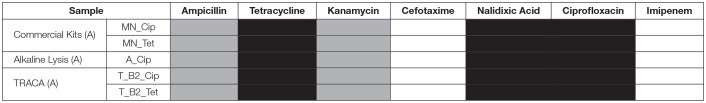
Transformants with identical resistance profiles from a disk diffusion assay.

*Along with a similar banding pattern, this indicates the strains are probably harboring the same plasmids. Black, Resistant; Gray, Intermediate; White, Susceptible; determined according to the (Clinical Laboratory Standards Institute, 2016)*.

*MN, Machery Nagel kit; A, Alkaline Lysis; T,TRACA*.

*Tet, selected on tetracycline; Cip, selected on ciprofloxacin*.

*B2, Higher band on gel; extracted and electroporated into E. coli DH5a*.

## Discussion

Plasmids isolated from complex samples have previously been examined using methods such as gradient gel resolution of PCR products, quantification of incompatibility groups using qPCR (Götz et al., 1996) or Southern blotting (Smalla et al., 2006). Recently, the study of plasmids involves the extraction of plasmid DNA followed by various sequencing approaches (Jørgensen et al., 2015). However, if multiple plasmids are present in a sample or if they are at low copy number, these won't be identified via sequencing due to the depth of current metagenomic sequencing technologies. Similarly, assembly is difficult with short-reads, especially if plasmids are present in low copy numbers or if the reads match to genomic DNA (De Toro et al., 2014). We performed this work to identify a method suitable for the extraction of plasmids harboring antibiotic resistance genes from complex broiler cecal samples, which could be transformed into a human pathogen, in this case *Escherichia coli*. This would then allow for further analysis, sequencing, and assembly of the plasmid in a well-defined bacterium. The variety of resistance plasmids obtained was determined by analysis of the banding patterns (shown in figures) and resistance profiles (shown in tables) of the transformants or transconjugants obtained.

In order to carry out studies on the overall resistance plasmid population present in a complex sample, the method to extract plasmid DNA must be optimized to give as best a representation as possible of the total resistance plasmids present. The first method we performed was a culture-dependent method on non-selective media. Another way of performing a more specific culture-dependent extraction would be to use selective agars. This would assist in the identification of which bacterial species a certain plasmid may have come from. However, this would introduce a bias to the results, as you would be choosing which agars and, hence, which bacteria to select. The main disadvantage of a culture-dependent method for complex samples is that only a small number of environmental bacteria can currently be cultured in the laboratory (Stewart, 2012). This means that by using only a culture-based method, it would greatly limit the number of plasmids isolated. Therefore, the representation of results from a culture-dependent study would give a limited view of the total resistance plasmidome. Our experiment did not yield any transformants on antibiotic plates, indicating that the plasmids present in the cultivable fraction did not harbor any resistance genes to the antibiotics tested.

The MoBio PowerSoil DNA Isolation kit is specifically designed to extract DNA from complex soil samples. This kit did not extract any plasmids harboring antibiotic resistance genes from our cecal sample as we failed to obtain any transformants, indicating it is more suitable for studies analyzing chromosomal DNA or fragmented DNA, rather than intact plasmid DNA. The Qiagen Plasmid Mini kit and the Machery-Nagel NucleoSpin Plasmid kit are both designed for the extraction of plasmids from bacterial culture. It seems that the cecal samples are too complex for these kits. The sample blocked the spin columns used in these kits, therefore little to no DNA was retrieved. Minimal success resulted with these commercial plasmid extraction kits, as few antibiotic resistance plasmids were obtained. It thus appears that these kits were not capable of dealing with the complexities associated with our samples.

The alkaline lysis method (Birnboim and Doly, 1979) is a common method of plasmid extraction, on which most commercial plasmid extraction kits are based. A benefit of using this method is that the chemicals used and their concentrations in the solutions can be decided upon and adapted for individual needs. There are standard protocols available for constituting resuspension, lysis and neutralization buffers, but, for example, additional enzymes can be added to the buffers. This type of adaptation is difficult with commercial kits as most do not share the components of their buffers. This is also why the alkaline lysis method and two commercial plasmid extraction kits were tested in this study, as they cannot be directly compared. However, this method still yielded few antibiotic resistance plasmids, even after additional modifications (as mentioned in Methods section) to the protocol.

The exogenous method of plasmid isolation is based on the capture of conjugative plasmids directly from a complex sample, via a recipient bacteria in biparental matings (Kyselková et al., 2016). This method captured resistance plasmids with different resistance profiles and was also the most consistent and not overly time-consuming. The disadvantages of this method are that it relies on the mobility of plasmids in the donor sample and the donor sample comprises an overnight culture of the total bacterial community. Therefore, the non-mobile plasmids present in the sample may not be captured with this method and the bacteria not capable of growth at the specific conditions will not be included as donors. However, many resistance plasmids are conjugative, and others can be mobile when assisted by a conjugative plasmid also residing in the same bacterial cell (Bennett, 2008). We are suggesting this method, not as a solution to all plasmid analysis problems, but rather as a first step in optimizing the analysis of antibiotic resistance plasmids from complex samples.

TRACA allowed for the acquisition of resistance plasmids from our sample but with similar banding patterns and resistance profiles. Therefore, it seems the expense associated with this method is not justified given the small variety of plasmids captured. Three of the four plasmids isolated using TRACA were also isolated using the alkaline lysis method and the Macherey-Nagel NucleoSpin Plasmid kit.

The multiple displacement amplification method allowed plasmids with the greatest range of resistance profiles to be obtained from our complex cecal samples. However, it should be noted that there were also difficulties and inconsistencies with this method. While good results were achieved using this method on Sample B (shown in results), many difficulties arose while carrying out the method on both Sample A and the control sample. The DNase step can be variable and time consuming, working well after one or two treatments at some times and not working after several more at other times. This also led to further downstream complications, as the more DNase treatments the sample was subjected to, the more salt that was present in the sample. This caused difficulties when performing electroporation, where salt concentration must be low. Therefore, the plasmid DNA isolated from both Sample A and control plasmid pEK499 did not transform into *E. coli*.

Plasmids now encode resistance to almost all classes of antibiotics currently in clinical use (Bennett, 2008). Therefore, the study of plasmids is crucial to fight the battle against antibiotic resistance that we are currently facing. Our comparative study shows the advantages and disadvantages of six methods for the extraction of plasmids harboring antibiotic resistance genes from complex broiler cecal samples, which can be applied to other complex environmental samples. This will assist researchers with the selection of the best method to use in their plasmid studies. Different gram-negative bacteria other than *E. coli* could be used for similar studies and the isolated plasmid DNA could be transformed into a gram-positive bacterium to further broaden the study. The exogenous plasmid isolation method was the best for obtaining a range of multi-drug resistance plasmids in a realistic timeframe with consistent results. However, even this method only resulted in a small range of resistance plasmids being isolated.

## Conclusion

Overall, the multiple displacement amplification method provided the greatest range of resistance plasmids from the investigated cecal samples. However, due to the inconsistencies of the results and the difficulties experienced with this method, it is not the ideal protocol to use when working with a large volume of samples under short deadlines. The commercial kits, alkaline lysis method and TRACA did not provide a wide range of resistance plasmids from our sample compared to the others tested. Therefore, the exogenous plasmid isolation method resulted in the widest range of resistance plasmids with ease of application and consistency across samples. While this method relies on the conjugative ability of the plasmids present, it is both an efficient (plasmids can be obtained in a short time-frame) and effective (a good range of plasmids can be acquired) method which worked with all of the cecal samples tested. Therefore, we recommend the exogenous plasmid isolation method when extracting antibiotic resistance plasmids of clinical relevance from a large number of complex samples.

## Ethics statement

Ethical approval was not required for this study. The sampling was performed by Alltech from a commercial farm. These sampling techniques were in line with national regulations about animal welfare ethics. All the animals were monitored throughout the study.

## Author contributions

SD performed all experiments, data analysis, and manuscript preparation. RM executed the data analysis and prepared manuscript. FW designed the study, executed the data analysis, and prepared the manuscript.

### Conflict of interest statement

The authors declare that the research was conducted in the absence of any commercial or financial relationships that could be construed as a potential conflict of interest.

## References

[B1] Arredondo-Alonso S. Willems R. J. Van Schaik W. Schürch A. C. (2017). On the (im)possibility of reconstructing plasmids from whole genome short-read sequencing data. Microb. Genom. 3:e000128. 10.1099/mgen.0.000128 29177087 PMC5695206

[B2] Bennett P. M. (2008). Plasmid encoded antibiotic resistance: acquisition and transfer of antibiotic resistance genes in bacteria. Br. J. Pharmacol. 153, S347–S357. 10.1038/sj.bjp.0707607 18193080 PMC2268074

[B3] Birnboim H. C. Doly J. (1979). A rapid alkaline extraction procedure for screening recombinant plasmid DNA. Nucleic Acids Res. 7, 1513–1523. 388356 10.1093/nar/7.6.1513 PMC342324

[B4] Clinical and Laboratory Standards Institute (2016). Performance Standards for Antimicrobial Susceptibility. CLSI Document M100S. Wayne, PA: Clinical and Laboratory Standards Institute.

[B5] De Toro M. Garcillán-Barcia M. P. De La Cruz F. (2014). Plasmid diversity and adaptation analyzed by massive sequencing of *Escherichia coli* plasmids. Microb. Spectr. 2, 219–235. 10.1128/microbiolspec.PLAS-0031-2014 26104438

[B6] Dib J. R. Wagenknecht M. Farías M. E. Meinhardt F. (2015). Strategies and approaches in plasmidome studies—uncovering plasmid diversity disregarding of linear elements? Front. Microbiol. 6:463. 10.3389/fmicb.2015.00463 26074886 PMC4443254

[B7] Götz A. Pukall R. Smit E. Tietze E. Prager R. Tschäpe H. (1996). Detection and characterization of broad-host-range plasmids in environmental bacteria by PCR. Appl. Environ. Microbiol. 62, 2621–2628. 8779598 10.1128/aem.62.7.2621-2628.1996 PMC168041

[B8] Halary S. Leigh J. W. Cheaib B. Lopez P. Bapteste E. (2010). Network analyses structure genetic diversity in independent genetic worlds. Proc. Natl. Acad. Sci. U.S.A. 107, 127–132. 10.1073/pnas.0908978107 20007769 PMC2806761

[B9] Hansen J. B. Olsen R. H. (1978). Isolation of large bacterial plasmids and characterization of the P2 incompatibility group plasmids pMG1 and pMG5. J. Bacteriol. 135, 227–238. 97269 10.1128/jb.135.1.227-238.1978 PMC224811

[B10] Jones B. V. Marchesi J. R. (2007). Transposon-aided capture (TRACA) of plasmids resident in the human gut mobile metagenome. Nat. Methods 4, 51–61. 10.1038/nmeth964 17128268

[B11] Jørgensen T. S. Kiil A. S. Hansen M. A. Sørensen S. J. Hansen L. H. (2015). Current strategies for mobilome research. Front. Microbiol. 5:750. 10.3389/fmicb.2014.00750 25657641 PMC4302988

[B12] Kav A. B. Benhar I. Mizrahi I. (2013). A method for purifying high quality and high yield plasmid DNA for metagenomics and deep sequencing approaches. *J. Microbiol*. Methods. 95, 272–279. 10.1016/j.mimet.2013.09.008 24055388

[B13] Klindworth A. Pruesse E. Schweer T. Peplies J. Quast C. Horn M.. (2013). Evaluation of general 16S ribosomal RNA gene PCR primers for classical and next-generation sequencing-based diversity studies. Nucleic Acids Res. 41:e1. 10.1093/nar/gks808 22933715 PMC3592464

[B14] Kyselková M. Chrudimsky T. Husník F. Chronákov,á A. Heuer H. Smalla K.. (2016). Characterization of *tet*(Y)-carrying lowGC plasmids exogenously captured from cow manure at a conventional dairy farm. FEMS Microbiol. Ecol. 92:fiw075. 10.1093/femsec/fiw07 27083193

[B15] Lynch M. D. Neufeld J. D. (2015). Ecology and exploration of the rare biosphere. Nat. Rev. Microbiol. 13, 217–229. 10.1038/nrmicro3400 25730701

[B16] Norman A. Riber L. Luo W. Li L. L. Hansen L. H. Sørensen S. J. (2014). An improved method for including upper size range plasmids in metamobilomes. PLoS ONE 9:e104405. 10.1371/journal.pone.0104405 25116381 PMC4130580

[B17] Oakley B. B. Kogut M. H. (2016). Spatial and temporal changes in the broiler chicken cecal and fecal microbiomes and correlations of bacterial taxa with cytokine gene expression. *Front. Vet*. Sci. 3:11 10.3389/fvets.2016.00011 PMC4759570 26925404

[B18] Perry J. A. Westman E. L. Wright G. D. (2014). The antibiotic resistome: what's new? *Curr. Opin*. Microbiol. 21, 45–50. 10.1016/j.mib.2014.09.002 25280222

[B19] Sentchilo V. Mayer A. P. Guy L. Miyazaki,1 R. Green Tringe S. Barry K.. (2013). Community-wide plasmid gene mobilization and selection. ISME J. 7, 1173–1186. 10.1038/ismej.2013.13 23407308 PMC3660673

[B20] Smalla K. Haines A. S. Jones K. Krogerrecklenfort E. Heuer H. Schloter M.. (2006). Increased abundance of IncP-1 plasmids and mercury resistance genes in mercury-polluted river sediments: first discovery of IncP-1 plasmids with a complex mer transposon as the sole accessory element. Appl. Environ. Microbiol. 72, 7253–7259. 10.1128/AEM.00922-06 16980416 PMC1636140

[B21] Sørensen S. Bailey M. Hansen L. H. Kroer N. Wuertz S. (2005). Studying plasmid horizontal transfer *in situ*: a critical review. Nat. Rev. Microbiol. 3, 700–710. 10.1038/nrmicro1232 16138098

[B22] Stewart E. J. (2012). Growing unculturable bacteria. J. Bacteriol. 194, 4151–4160. 10.1128/JB.00345-12 22661685 PMC3416243

[B23] Tamminen M. Virta M. Fani R. Fondi M. (2012). Large-scale analysis of plasmid relationships through gene-sharing networks. Mol. Biol. Evol. 29, 1225–1240. 10.1093/molbev/msr292 22130968

[B24] Thomas C. M. Nielsen K. M. (2005). Mechanisms of, and barriers to, horizontal gene transfer between bacteria. Nat. Rev. Microbiol. 3, 711–721. 10.1038/nrmicro1234 16138099

[B25] Warburton P. J. Allan E. Hunter S. Ward J. Booth V. Wade W. G.. (2011). Isolation of bacterial extrachromosomal DNA from human dental plaque associated with periodontal disease, using transposon-aided capture (TRACA). FEMS Microbiol. Ecol. 78, 349–354. 10.1111/j.1574-6941.2011.01166.x 21711368 PMC3263338

