# Supplementary material for: A Comparison of Methods for the Extraction of Plasmids Capable of Conferring Antibiotic Resistance in a Human Pathogen From Complex Broiler Cecal Samples
Source: Front Microbiol. 2018 Aug 13;9:1731. doi: 10.3389/fmicb.2018.01731 (PMC6100392; doi:10.3389/fmicb.2018.01731)
Supplement: Supplementary file 1 [file Data_Sheet_1.pdf]

Supplementary Material

A Comparison of Methods for the Extraction of Plasmids Capable of Conferring Antibiotic Resistance in a Human Pathogen from Complex Broiler Cecal Samples

Sarah Delaney*, Richard Murphy, Fiona Walsh

*** Correspondence:** Sarah Delaney: sarah.delaney@mu.ie

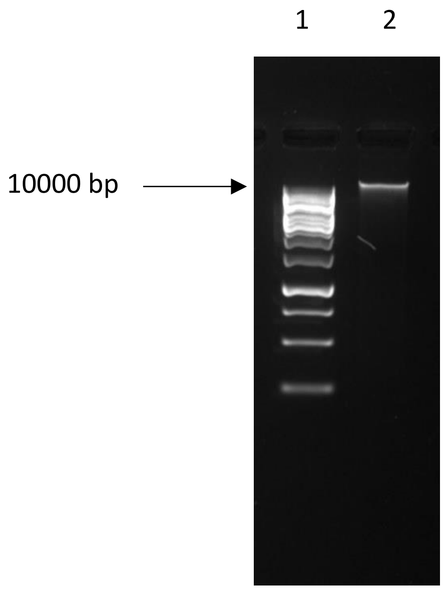

Supplementary Figure 1. Agarose gel image of the pEK499 plasmid extracted from the cultured *E. coli* using the culture dependent method.

1= 1 kb ladder; **2**= DNA extracted from *E. coli* harbouring the pEK499 plasmid.

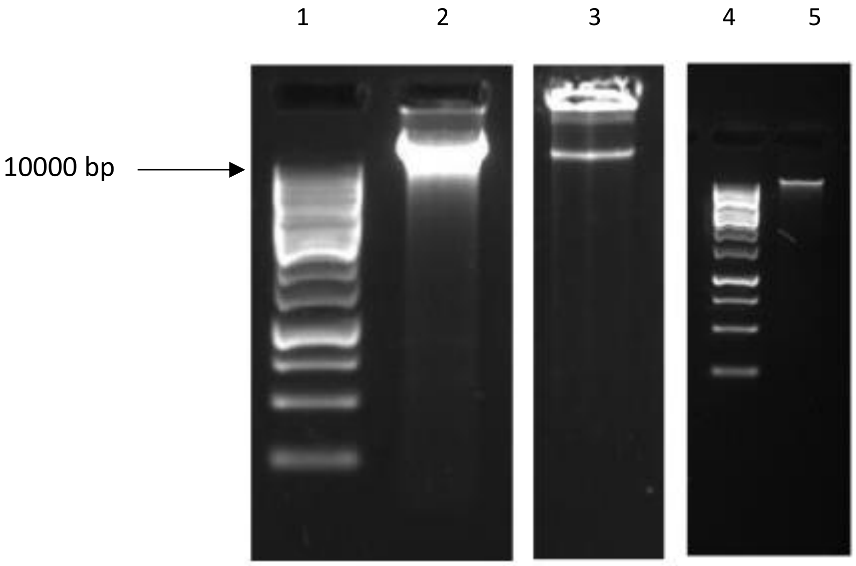

Supplementary Figure 2. Agarose gel image of control plasmid pEK499 extracted using the commercial kits.

1= 1 kb ladder; **2** = control plasmid pEK499 extracted using the MoBio PowerSoil DNA Isolation Kit; **3 =** control plasmid pEK499 extracted using the Qiagen Plasmid Mini Kit; **4**= 1 kb ladder; **5** = control plasmid pEK499 extracted using the Macherey-Nagel NucleoSpin Plasmid Kit.

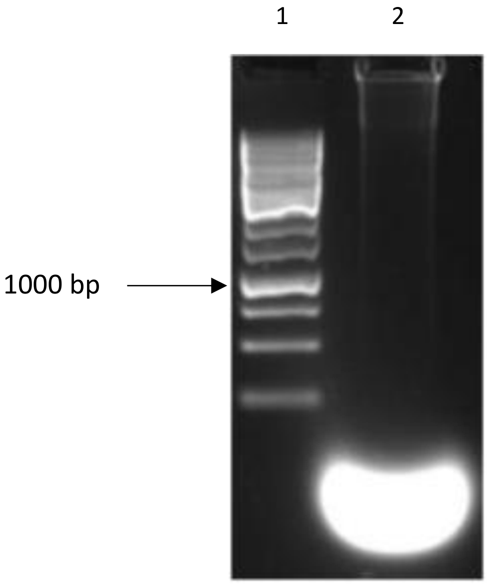

Supplementary Figure 3. Agarose gel image of pEK499 extraction using the alkaline lysis method.

1= 1 kb Ladder; **2**= control plasmid pEK499 extracted using the alkaline lysis method.

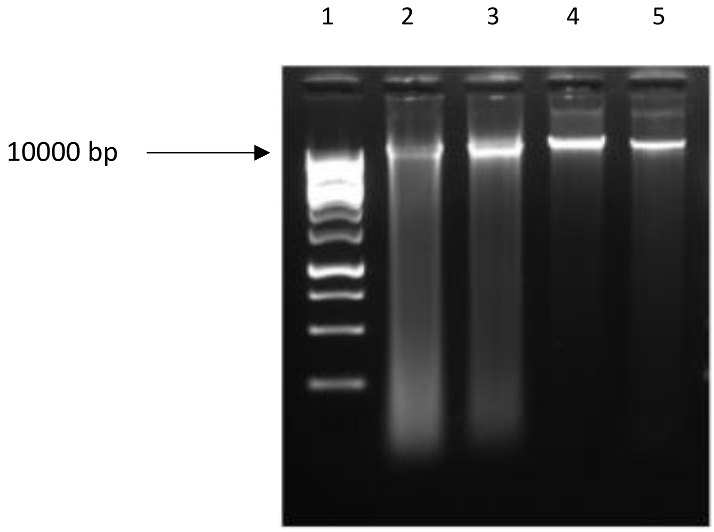

Supplementary Figure 4. Agarose gel image of pEK499 isolated using the exogenous method.

1= 1 kb ladder and control plasmid pEK499 extracted using the exogenous plasmid isolation method. Plasmid DNA extracted from transformants selected on agar plates containing: **2**= ampicillin 32 mg/L; **3**= tetracycline 16 mg/L; **4=** kanamycin 25 mg/L; and **5=** ciprofloxacin 4 mg/L.
